# Subversion of the Endocytic and Secretory Pathways by Bacterial Effector Proteins

**DOI:** 10.3389/fcell.2018.00001

**Published:** 2018-01-24

**Authors:** Mary M. Weber, Robert Faris

**Affiliations:** Department of Microbiology and Immunology, University of Iowa, Iowa City, IA, United States

**Keywords:** Coxiella, Brucella, Salmonella, Legionella, Chlamydia, Orientia, secreted effector, vesicle trafficking

## Abstract

Intracellular bacteria have developed numerous strategies to hijack host vesicular trafficking pathways to form their unique replicative niches. To promote intracellular replication, the bacteria must interact with host organelles and modulate host signaling pathways to acquire nutrients and membrane for the growing parasitophorous vacuole all while suppressing activation of the immune response. To facilitate host cell subversion, bacterial pathogens use specialized secretion systems to deliver bacterial virulence factors, termed effectors, into the host cell that mimic, agonize, and/or antagonize the function of host proteins. In this review we will discuss how bacterial effector proteins from *Coxiella burnetii, Brucella abortus, Salmonella enterica* serovar Typhimurium, *Legionella pneumophila, Chlamydia trachomatis*, and *Orientia tsutsugamushi* manipulate the endocytic and secretory pathways. Understanding how bacterial effector proteins manipulate host processes not only gives us keen insight into bacterial pathogenesis, but also enhances our understanding of how eukaryotic membrane trafficking is regulated.

Obligate and facultative intracellular bacteria have developed numerous methods to hijack host membranes to promote uptake, survival, and intracellular replication. Following uptake, intracellular pathogens must engage host organelles and subvert host defense mechanisms to establish their unique intracellular niches. To facilitate interactions with the host, many pathogenic bacteria deliver bacterial virulence proteins, termed effectors, into the host cell using specialized secretion systems. These proteins traffic to distinct subcellular locations within the host cell (Weber et al., 2013, 2016a; Beyer et al., 2017; Miller et al., 2017) or decorate the bacterial vacuole (Larson et al., 2015; Weber et al., 2015). Within the host cell, effector proteins work to promote acquisition of nutrients, redirect host membranes to the growing vacuole, and promote host cell viability to provide adequate time for bacterial replication (Larson et al., 2013; Weber et al., 2017). To achieve these feats, many effector proteins utilize “eukaryotic-like domains” that mimic the form and/or function of host proteins (Delevoye et al., 2008; Chen et al., 2010; Beyer et al., 2015), which in turn promotes manipulation of the targeted host cell pathway. In this review, we will discuss some of the well-characterized strategies used by intracellular bacteria to disrupt normal host vesicular trafficking pathways and touch on cutting edge research unraveling novel mechanisms used by pathogens to usurp the host. Specifically, our emphasis will be on how bacterial secreted effector proteins subvert the endocytic and secretory pathways to promote formation of their intracellular niche.

The eukaryotic cell can monitor its environment and acquire essential nutrients through a process called endocytosis in which extracellular particles or molecules are engulfed by the plasma membrane and trafficked through the endocytic pathway. In addition, endocytic trafficking pathways play an integral role in recycling or degrading receptors, lipid membranes, or extracellular fluid that is internalized via clathrin-dependent or clathrin-independent pathways (Jovic et al., 2010). Generally, internalized vesicles are released from the plasma membrane by scission and fuse with early endosomes (Gautreau et al., 2014), which serve as a sorting station for cargo to be recycled back to the plasma membrane or undergo degradation in late endosomes and lysosomes (Jovic et al., 2010; Figure 1).

**Figure 1:**
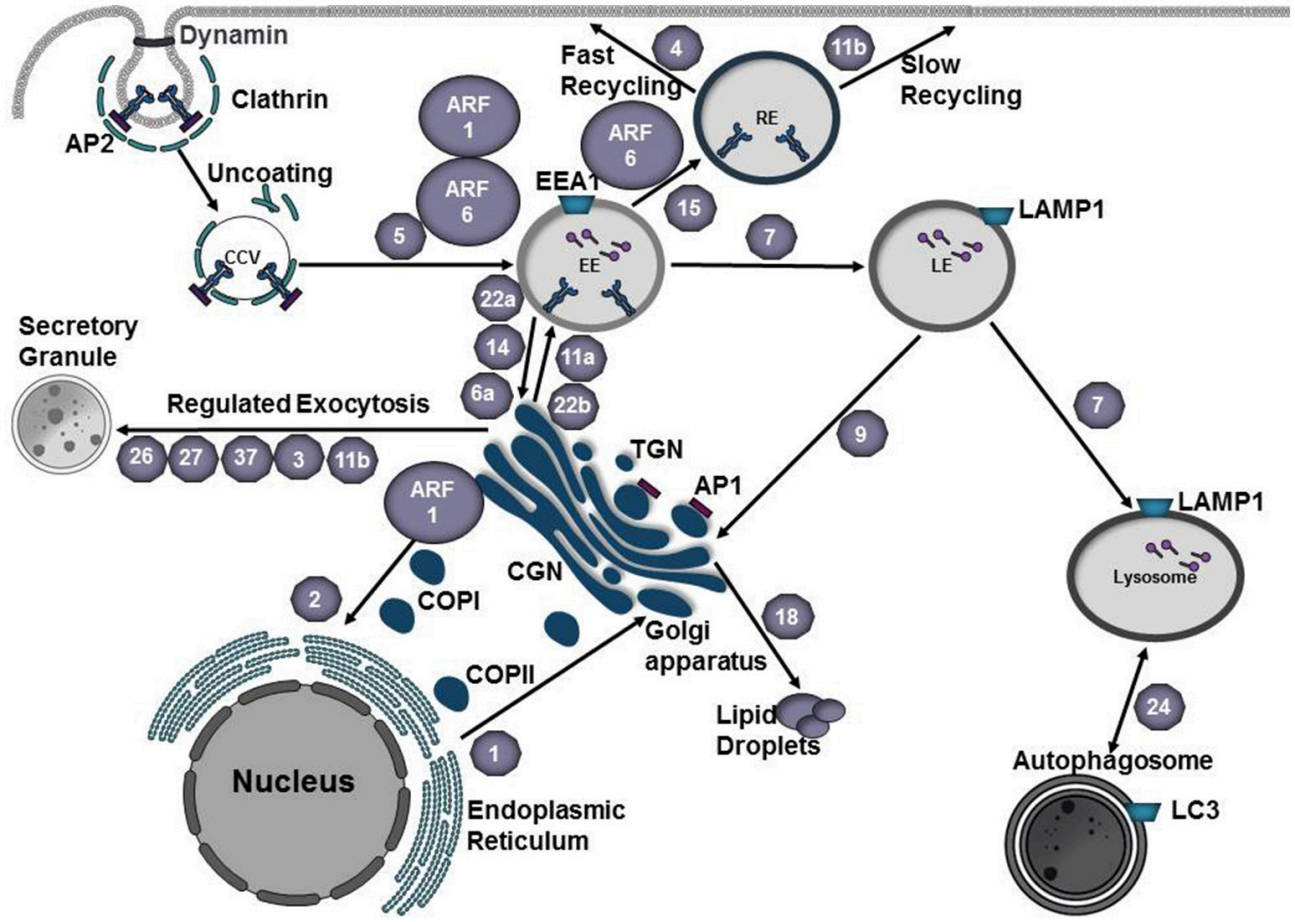
Vesicle trafficking pathways of the endocytic and secretory pathways. Small GTPases of the Arf family recruit protein coats to the donor membrane to initiate vesicle budding. Clathrin coated vesicles are generated at the plasma membrane or *trans-*Golgi network (TGN) and mediate cargo transport to endosomes. Adaptor proteins, such as AP1 and AP3, recognize signal sequences in the C-terminal tails of transmembrane proteins and participate in cargo selection. Coat complex protein I (COPI) and coat complex protein II (COPII) mediate transport from the Golgi to the endoplasmic reticulum (ER) or from the ER to the Golgi, respectively. At the plasma membrane, vesicle scission is mediated by dynamin and vesicles are subsequently transported to their destination along cytoskeletal tracks. Rab GTPases (gray hexagons) play a key role in regulating vesicle transport along the endocytic and secretory pathways. Rab5 mediates fusion of endocytic vesicles to form the early endosome (EE). Maturation into a late endosome (LE) requires Rab conversion from Rab5 to Rab7. Cargo destined for degradation undergoes fusion with lysosomes. Rab24 mediates formation of autophagosomes, which can subsequently fuse with lysosomes to create autolysosomes. Traffic between EE and recycling endosome (RE) is mediated by Rab15. Rab4 and Rab11b regulate fast and slow recycling, respectively. Rab1 mediates ER-Golgi traffic whereas Rab2 regulates retrograde traffic from the Golgi-ER. Regulated exocytosis by secretory granules involves Rab26, Rab27, Rab37, Rab3, or Rab11b. Transport between the EE and Golgi or the LE and Golgi is mediated by Rab22a, Rab14, Rab6a, or Rab9, respectively. Cargo from the TGN is transported to the plasma membrane via fusion with EEs and this process is mediated by Rab22b or Rab11a. Rab18 regulates the formation of lipid droplets. EE early endosome, LE late endosome, RE recycling endosome TGN *trans*-Golgi Network, CGN *cis*-Golgi Network, COPI coat protein I, COPII coat protein II, CCV clathrin-coated vesicle, AP1 adaptor protein 1, AP2 adaptor protein 2.

Rab proteins regulate vesicle budding, transport, tethering, and fusion of transport vesicles as they move from donor to acceptor compartments (Cai et al., 2007). Rab proteins cycle between an inactive GDP-bound or active GTP-bound state. Binding of GTP induces a conformational change in the switch regions that flank the nucleotide-binding site, exposing protein-protein interaction regions necessary for binding of Rab effector proteins. The nucleotide-bound state of Rab proteins is determined by guanine nucleotide exchange factors (GEFs) that activate Rabs by promoting GDP to GTP exchange and GTPase activating proteins (GAPs) that inactivate Rabs by catalyzing the hydrolysis of GTP (Hutagalung and Novick, 2011).

Rab5 on the early endosome is activated by the guanine exchange factor (GEF) Rabex-5 (Horiuchi et al., 1997). Activation of Rab5 results in recruitment of hVPS34, a PI3-kinase that produces PtdIns(3)P, which serves as a signal for recruitment of effector proteins such as sorting nexins (Cozier et al., 2002), EEA1 (Lawe et al., 2000), or Rabenosyn-5 (Nielsen et al., 2000). Progression to a late endosome requires exchange of Rab5 for Rab7 (Figure 1). In this process Vps39, a GEF for Rab7 (Rink et al., 2005) is recruited to the endosome and activates Rab7 whereas Rab5 is inactivated by RabGAP5 (Haas et al., 2005). Activated Rab7 mediates attachment of the late endosome to the dynein-dynactin complex (Cantalupo et al., 2001; Jordens et al., 2001), which promotes translocation of the late endosome to the microtubule organizing center (MTOC) via microtubules. Here the late endosome fuses with the lysosome, the lumen acidifies via v-ATPases and LAMP1, hydrolases and proteases are activated, and cargo is degraded (Saftig and Klumperman, 2009; Figure 1).

In eukaryotes, secretory proteins (ex. transmembrane proteins and lysosomal proteins) are synthesized in the rough endoplasmic reticulum (ER), delivered to the ER-Golgi intermediate compartment (ERGIC), modified as they move through the Golgi, and are ultimately packaged into transport vesicles for delivery to their final destination (ex. plasma membrane, endosome, lysosome; Kim, 2016). Transport of cargo between organelles involves membrane-bound vesicles that bud from a donor membrane and fuse with an acceptor organelle. These transport vesicles are divided into three classes based on their protein coat: clathrin-coated vesicles (CCVs) originate at the plasma membrane or *trans*-Golgi face and fuse with endosomes or lysosomes; COPII-coated vesicles move cargo from the ER to the Golgi, whereas COPI-coated vesicles move between Golgi cisternae or from the cis-Golgi back to the ER (retrograde transport; Gomez-Navarro and Miller, 2016; Figure 1). Budding is initiated by the recruitment of specific coat complexes from the cytosol to the donor membrane by small GTPases of the Arf1/Sar1 family (Springer et al., 1999) or through an interaction with organelle-specific phosphoinositide lipid (Cai et al., 2007). Coat proteins, along with adaptor proteins or accessory factors, recognize specific signal sequences in the cytoplasmic domain of cargo (Cai et al., 2007). Multiple cargo adaptors are capable of interacting with the same coat scaffold and although scaffold proteins are able to act in a multitude of subcellular locations, cargo adaptor/accessory proteins are organelle specific. Following budding from the donor membrane, transport vesicles move along actin or microtubule tracks using the molecular motors myosin, dynein, or kinesin (Hammer and Wu, 2002; Cai et al., 2007). Prior to fusion, transport vesicles interact with tethers that include long coil-coiled proteins or multi-subunit complexes (Cai et al., 2007). Activated Rab GTPases and their effectors play an integral role in recruiting tethers to the appropriate location and participate in vesicle uncoating (Semerdjieva et al., 2008; Ostrowicz et al., 2010; Balderhaar et al., 2013). Fusion of the transport vesicle with the target organelle is mediated by pairing of SNAREs on the transport vesicle (v-SNARE) with those on the target organelle (t-SNARE).

Intracellular bacteria have evolved highly efficient methods for entering the host cell and subverting endocytic trafficking pathways to establish their intracellular niche. Following initial attachment to a host cell, bacteria either commandeer host receptors to promote bacterial entry or secrete bacterial effector proteins that modulate host cell signaling pathways to promote uptake (Asrat et al., 2014). Phagosome acidification is a common strategy used by the host to control bacterial infection and aids in breakdown of cellular components. However, some vacuolar pathogens have developed sophisticated strategies to usurp trafficking through the endocytic pathway while others embrace vacuole acidification to complete intracellular replication.

## Coxiella burnetii

*Coxiella burnetii* is an obligate intracellular bacterium that is the etiological agent of acute and chronic Q fever in humans. Acute Q fever is typically acquired through inhalation of contaminated soil or animal products, resulting in an acute flu-like illness that usually resolves in 1–2 weeks. However, in some instances chronic Q fever can occur and generally presents as endocarditis or hepatitis (van Schaik et al., 2013).

**Figure 2:**
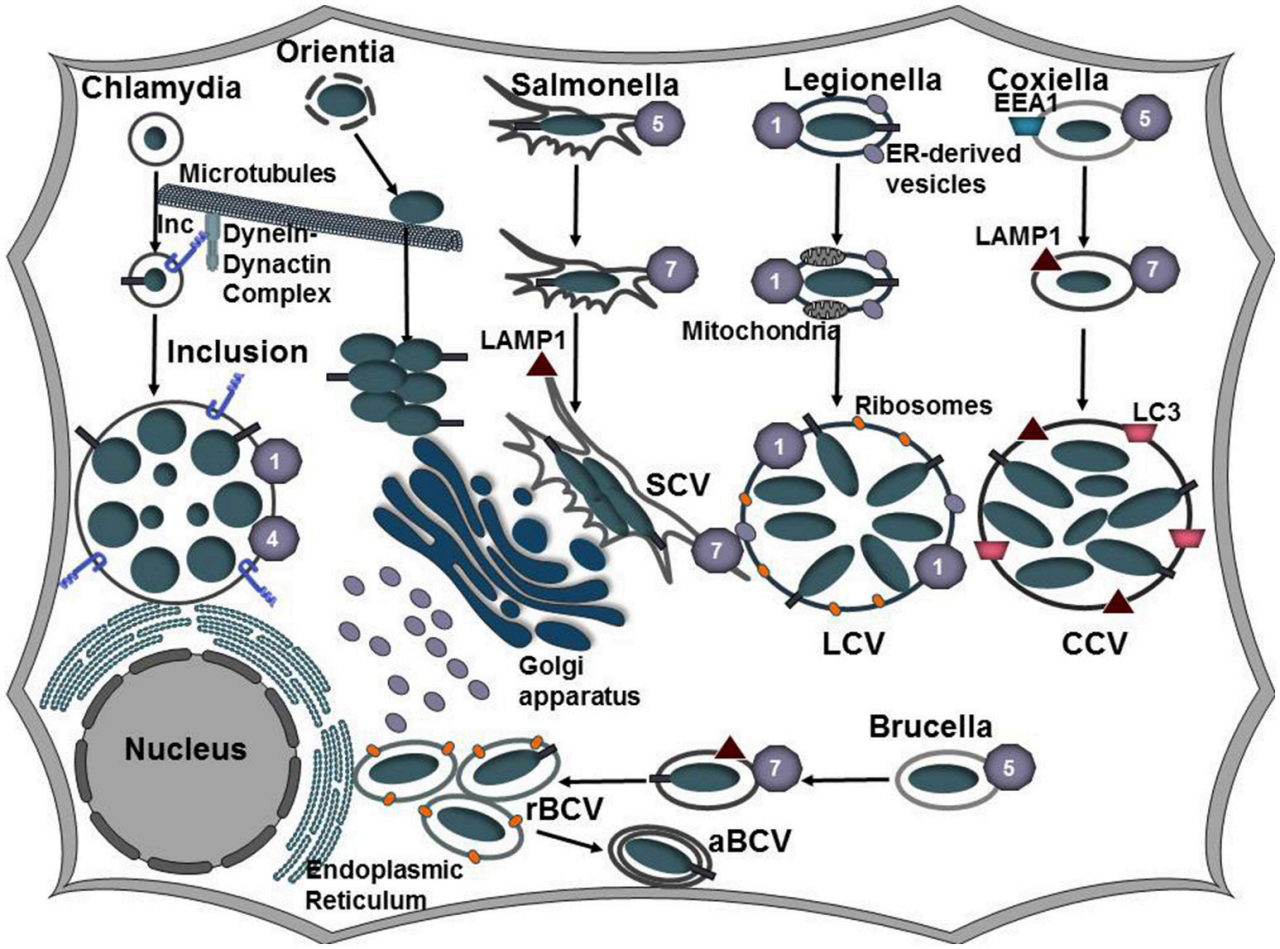
Establishing a replicative niche. Following uptake by a host cell, intracellular bacteria manipulate the endocytic and secretory pathways of the host cell to establish a replicative niche. *Coxiella, Salmonella*, and *Brucella* associate with the endocytic pathways as evident by Rab5, Rab7, and LAMP1 that decorate the *Coxiella*-containing vacuole (CCV), *Salmonella*-containing vacuole (SCV), and *Brucella*-containing vacuole (BCV), respectively. As the CCV matures, it undergoes fusions with autophagosomes and acquires LC3. Vacuolar acidification induces expression of the *C. burnetii* Dot/Icm and the *Brucella* VirB type IV secretion systems. Following transient vacuolar acidification, the BCV is redirected to and fuses with the endoplasmic reticulum to form the rBCV. Some bacteria undergo interactions with autophagosomes (aBCV) as a possible exit mechanism. *Salmonella* replicates in the peri-Golgi region and induces the formation of tubular membranes, referred to as *Salmonella*-induced filaments (SIFs) that are enriched in LAMP1. *L. pneumophila* secretes Dot/Icm effector proteins to bypass the endocytic pathway and instead redirects ER-derived vesicles to the LCV. *C. trachomatis* also evades the endocytic pathway and inhibits fusions with lysosomes. Modification of the inclusion membrane by incorporation of type III secreted effector proteins, termed inclusion membrane proteins (Incs), promotes trafficking of the inclusion along microtubules to the microtubule organizing center (MTOC). The bacteria undergo conversion from the elementary body (EB) to the replicative body (RB) and the bacteria replicate by binary fission. Following internalization, *Orientia tsutsugamushi* escapes the host-derived vacuole and traffics along microtubules to replicate in the cytosol juxtaposed to the ER and Golgi apparatus.

*Coxiella burnetii* traffics through the default endocytic pathway to establish the *Coxiella*-containing vacuole (CCV; Figure 2). Within a few hours of infection, the CCV is decorated with the autophagosomal makers LC3 (Romano et al., 2007), Beclin1 (Vázquez and Colombo, 2009), and p62 (Winchell et al., 2014). Engagement of the autophagic machinery appears to benefit the pathogen, presumably by serving as a source of membrane for the growing CCV. Within 1–2 h post-infection, the CCV acidifies and lysosomal markers such as cathepsin D and acid phosphatase localize within the CCV. Vacuolar acidification promotes intracellular replication and activation of the Dot/Icm type IV secretion system (T4SS) which results in effector translocation around 8 h post-infection (Heinzen et al., 1996; Newton et al., 2013). *C. burnetii* is uniquely adapted to reside in this acidic autophagoloysosome-like compartment and as such does not actively manipulate early trafficking events like other pathogens that must avoid lysosomal fusion for intracellular survival. Between 8 h and 2 d post-infection the CCV undergoes homotypic fusion with other CCVs and heterotypic fusion with endocytic, lysosomal, and autophagosomal vesicles. Ultimately the *Coxiella*-containing vacuole will expand to occupy the majority of the host cytoplasmic space (Larson et al., 2016). Over 130 *C. burnetii* Dot/Icm type IV secreted effectors have been identified (Chen et al., 2010; Carey et al., 2011; Voth et al., 2011; Weber et al., 2013; Larson et al., 2015). Deletion of single effector proteins in the comparative *L. pneumophilia* model does not generally diminish virulence, whereas many *C. burnetii* effectors have been identified as essential for intracellular replication, suggesting there is less redundancy among *C. burnetii* effectors (Weber et al., 2013; Martinez et al., 2014; Newton et al., 2014; Larson et al., 2015). While advances in *Coxiella* genetics have significantly advanced our understanding of potential effector function, little is known about how *C. burnetii* hijacks host vesicular pathways and the molecular function of most of the Dot/Icm secretion substrates remain unknown.

**Figure 3:**
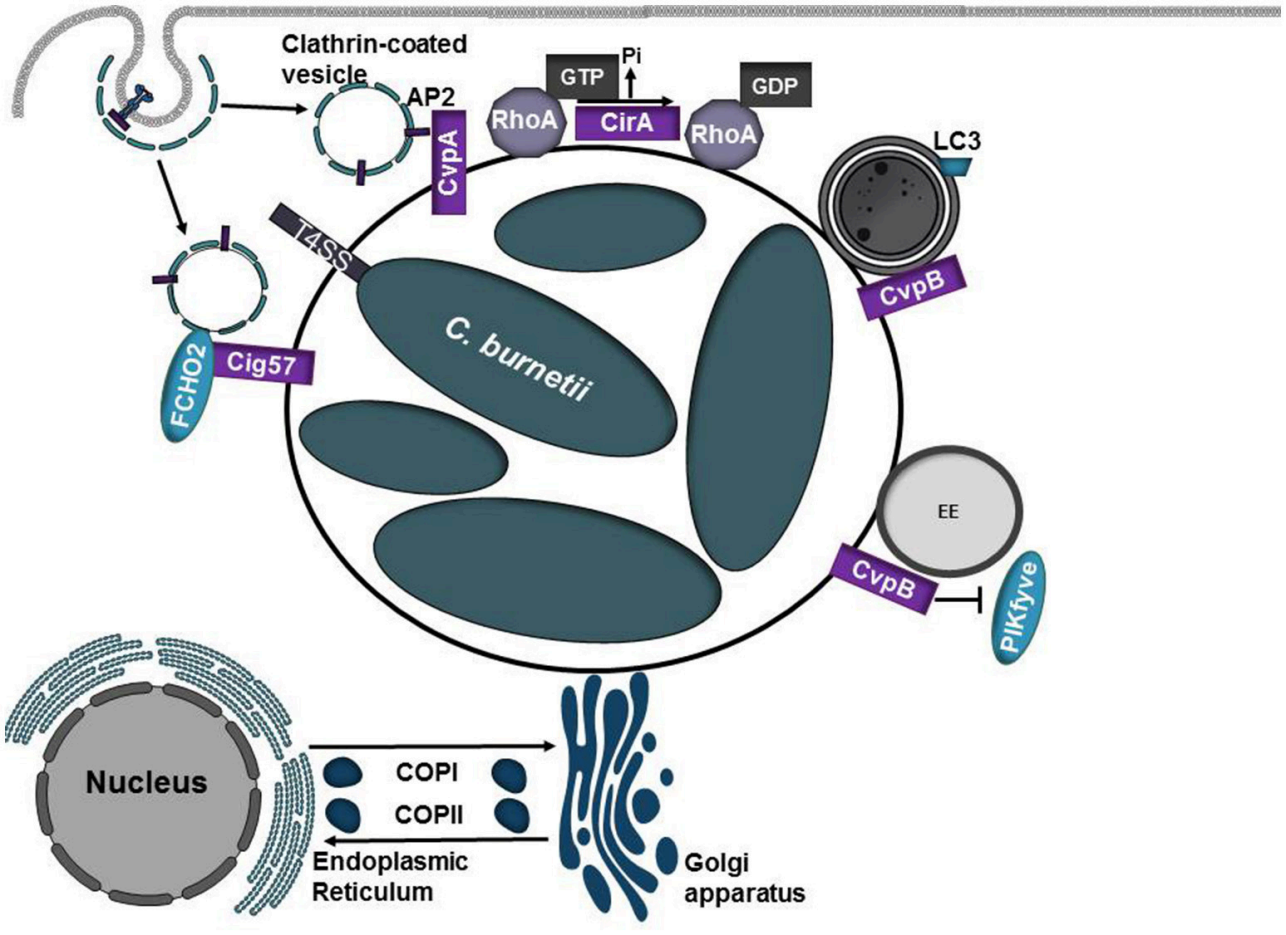
Diverse roles of *C. burnetii* T4SS effector proteins. *Coxiella burnetii* replicates in an acidic phagolysosome-like compartment termed the *Coxiella-*containing vacuole (CCV). Acidification of the CCV promotes activation of the Dot/Icm type IV secretion system (T4SS) which translocates over 130 proteins into the eukaryotic host cell. As the CCV matures, it undergoes fusions with autophagosomes and acquires the autophagosomal maker LC3. CvpA and Cig57 modulate clathrin-dependent vesicular transport pathways through interactions with adaptor protein 2 (AP2) or FCHO_2_, respectively. CirA acts as a GTPase activating protein (GAP) for RhoA. CvpB/Cig2 promotes CCV expansion by targeting endosomes and autophagosomes. CvpB/Cig2 interferes with PI 5-kinase PIKfyve access to early endosomes, resulting in increased levels of PI(3)P on CCVs.

Both CvpA and Cig57 modulate clathrin-dependent vesicle trafficking pathways by binding clathrin adaptor proteins or the clathrin accessory protein FCHO2, respectively (Larson et al., 2013; Latomanski et al., 2016; Figure 3, Table 1). Depletion of clathrin or mutation of CvpA or Cig57 reduces *Coxiella* replication and CCV expansion (Larson et al., 2013; Latomanski et al., 2016), suggesting that manipulation of clathrin-dependent vesicle transport pathways may serve as a source of membrane for the growing CCV. Surprisingly, FCHO2 is not recruited to the CCV nor does depletion of FCHO2 significantly reduce *C. burnetii* replication, however siRNA knockdown of FCHO2 significantly reduces clathrin recruitment to the CCV (Latomanski et al., 2016). Collectively these results suggest a model in which Cig57 engages FCHO2 at the plasma membrane to initiate formation of CCVs to promote biogenesis and maturation of the CCV.

**Table 1:** Bacterial effectors and their host targets.

**Bacterial species**	**Secreted effector**	**Host target**	**Function**	**Consequence**	**References**
*Coxiella burnetii*	CvpA	AP2, clathrin		Endocytic sorting signals in CvpA bind AP2. Modulates clathrin-dependent vesicle transport on the CCV membrane.	Larson et al., 2013
	Cig57	FCHO2		Modulates clathrin-dependent vesicle transport by interacting with FCHO2 to promote biogenesis of CCV.	Latomanski et al., 2016
	CirA	RhoA	GAP for RhoA	GAP for RhoA. Promotes cytoskeletal rearrangements to promote vesicle delivery to CCV.	Weber et al., 2016b
	CvpB/Cig2	PI(3)P, PS		Binds PI(3)P and PS and interferes with PI 5-kinase PIKfyve access to early endosomes, increases level of PI(3)P on CCV. Triggers vacuolation at early endosomes and at autophagosomes it stabilizes the autophagic machinery, promotes CCV expansion.	Newton et al., 2014; Martinez et al., 2016
*Brucella abortus*	RicA	Rab2		Interacts with and recruits Rab2 to the BCV	de Barsy et al., 2011
	BspB	COG complex (COG1-5, COG7)		Interacts with COG complex to redirect Golgi-derived vesicles to the BCV	Miller et al., 2017
*Salmonella enterica* serovar Typhimurium	SopB	PI(4,5)P_2_, PS	Phosphoinositide phosphatase	Phosphoinositide phosphatase that modulates Rab recruitment through mediating charge on the SCV	Mallo et al., 2008; Bakowski et al., 2010
	SopD2	Rab7, Rab32	GAP for Rab32	Binds Rab7 and prevents interactions with RILP and FYCO1	D'Costa et al., 2015; Spanò et al., 2016
	SseF	ACBD3		Tethers the SCV to the Golgi	Yu et al., 2016
	SseG	ACBD3		Tethers the SCV to the Golgi	Yu et al., 2016
	SifA	SKIP		Sequesters Rab9 through SifA-SKIP interactions to reduce lysosome potency by interfering with retrograde trafficking of MPR to the *trans-*Golgi	Boucrot et al., 2005; McGourty et al., 2012
*Legionella pneumophilia*	VipD	Rab5, Rab22	Binds Rab5 and Rab22 to prevent recruit of Rab effectors	Prevents endocytic maturation	Ku et al., 2012; Lucas et al., 2014
	SidM/DrrA	Rab1	GEF and AMPylator for Rab1	Promotes fusion between the LCV and ER-derived vesicles	Machner and Isberg, 2006; Murata et al., 2006; Müller et al., 2010; Arasaki et al., 2012
	SidD	Rab1	deAMPylase for Rab1	Removes AMP moiety from Rab1, allows for deactivation by GAPs	Neunuebel et al., 2011; Tan and Luo, 2011
	LepB	Rab1	GAP for Rab1	Inactivates Rab1 through GTP hydrolysis	Ingmundson et al., 2007
	AnkX	Rab1, Rab35	PCylation of Rab1	Adds a PC moiety to Rab1, prevents association with effectors and deactivation by GAPs	Tan et al., 2011; Goody et al., 2012
	Lem3	Rab1	dePCylation of Rab1	Remove PC moiety, allows for deactivation by GAPs	Tan et al., 2011
	LidA	Rab1, Rab6, Rab8		Cooperates with SidM/DrrA, enhances fusion of LCV with ER-derived vesicles by prolonging activation of Rab1	Machner and Isberg, 2006
	RalF	Arf1	GEF for Rab1	Activates and recruits Arf1 to the LCV to promote recruitment and recycling of ER-derived vesicles	Nagai et al., 2002
	SidE	Rab1, Rab6A, Rab30, Rab33b, RTN4	Ubiquitin ligase and deubiquitylase, phosphodiesterase	Ubiquitination of Rab GTPases, rearrangement of tubular ER	Qiu et al., 2016; Kotewicz et al., 2017
	SdeA	Rab1, Rab6A, Rab30, Rab33b, RTN4	Ubiquitin ligase and deubiquitylase, phosphodiesterase	Ubiquitination of Rab GTPases, rearrangement of tubular ER	Qiu et al., 2016; Kotewicz et al., 2017
	SdeB	Rab1, Rab6A, Rab30, Rab33b, RTN4	Ubiquitin ligase and deubiquitylase, phosphodiesterase	Ubiquitination of Rab GTPases, rearrangement of tubular ER	Qiu et al., 2016; Kotewicz et al., 2017
	SdeC	Rab1, Rab6A, Rab30, Rab33b, RTN4	Ubiquitin ligase and deubiquitylase, phosphodiesterase	Ubiquitination of Rab GTPases, rearrangement of tubular ER	Qiu et al., 2016; Kotewicz et al., 2017
	RidL	VPS29, PtdIns(3)P		Binds PtdIns(3)P and retromer subunit VPS29 to promote formation of nonlysosomal replicative compartment	Finsel et al., 2013
*Chlamydia trachomatis*	IncA	VAMP3, VAMP7, VAMP8		Homotypic fusion of inclusions, inhibits fusions with lysosomes	Delevoye et al., 2008; Ronzone and Paumet, 2013; Ronzone et al., 2014; Weber et al., 2016c
	CT229 (CpoS)	Rab1a,b Rab2b, Rab34, Rab6a,b Rab8a,b Rab10, Rab14, Rab35, Rab18, Rab33b, Rab4		CT229 is required for Rab recruitment to the inclusion, CT229 mutants are defective in intracellular replication and inclusion development	Rzomp et al., 2006; Mirrashidi et al., 2015; Sixt et al., 2017; Weber et al., 2017
	CT813 (InaC)	Arf1, Arf4, Arf5, 14-3-3	Activates Arf1	Interacts with Arf GTPase to control Golgi ministack positioning around the inclusion	Kokes et al., 2015; Wesolowski et al., 2017
	IncE	SNX5/6		Binds and recruits SNX5/6 to the inclusion, increases inclusion membrane tabulation, disrupts retromer trafficking	Mirrashidi et al., 2015; Elwell et al., 2017
	IncD	CERT		Binds CERT to promote import of ceramide to the inclusion	Derré et al., 2011
	CT619	Hrs, Tsg101		Unknown	Vromman et al., 2016
	CT620	Hrs		Unknown	Vromman et al., 2016
	CT621	Hrs		Unknown	Vromman et al., 2016
	CT711	Hrs		Unknown	Vromman et al., 2016
	CT712	Hrs		Unknown	Vromman et al., 2016
*Orientia tsutsugamushi*	Ank9	COPB2, SKP1		Interacts with COPB2 at Golgi to co-opt COPI-mediated retrograde trafficking to the ER, induces ATF4-mediated UPR to disrupt protein secretion	Beyer et al., 2017

Another essential type IV effector protein, CirA, can function as a GAP for RhoA and is hypothesized to promote cytoskeletal rearrangements to promote redirection of host vesicles to the growing CCV (Weber et al., 2016b). CirA is predicted to encode several arginine-like finger motifs that support the observation that CirA acts as GTPase activating protein. However, whether these motifs and CirA's associated GAP activity are necessary in the context of infection has yet to be determined.

Following infection, vacuoles containing replication-competent bacteria undergo homotypic fusions to form a single replicative niche. Screening of transposon mutant libraries has identified several *C. burnetii* effector proteins that are required for homotypic fusion of CCVs (Newton et al., 2014). Transposon insertion into CvpB (also referred to as Cig2) results in multiple CCVs per cell, which are defective in homotypic fusion and are unable to form the characteristic single CCV per cell. Similar defects in CCV fusion are noted when the essential autophagy proteins ATG5 and ATG12 are silenced, suggesting that autophagy is important for homotypic fusion of CCVs and CvpB/Cig2 may target this pathway (Newton et al., 2014). Vacuoles generated by CvpB/Cig2 mutants display diminished recruitment of LC3, however defects in host autophagic flux are not observed (Newton et al., 2014). CvpB/Cig2 binds PI(3)P and PS and interferes with PI 5-kinase PIKfyve access to early endosomes, resulting in increased levels of PI(3)P on CCVs (Martinez et al., 2016). At early endosomes, CvpB/Cig2 triggers vacuolation to promote CCV expansion and at autophagosomes it stabilizes the autophagic machinery to promote homotypic fusion of CCVs (Martinez et al., 2016).

## Brucella abortus

*Brucella* spp. are Gram-negative facultative intracellular bacteria that are the causative agents of brucellosis, a zoonotic disease of global importance. *Brucella* spp. infect a variety of mammalian species and the bacteria has a tropism for the reproductive system, causing abortion and sterility in animals. Brucellosis in humans is caused by *Brucella abortus, Brucella melitensis*, or *Brucella suis* and the disease is characterized by recurrent fever, endocarditis, neurological symptoms, and chronic fatigue (Celli, 2015).

*Brucella abortus* similarly traffics through the endocytic pathway and the *Brucella*-containing vacuole (BCV) associates with early and late endosomal compartments (Starr et al., 2008; Figure 2). Limited interactions with lysosomes allows transient acidification of the endosomal compartment, which is hypothesized to promote activation of the VirB type 4 secretion system (T4SS; Boschiroli et al., 2002). However, this interaction is transitory and the BCV uses ER-derived vesicles to form a replication-competent vacuole termed the rBCV. While the exact mechanism is unknown, effector proteins secreted by the VirB T4SS appear to prevent accumulation of lysosomal markers on the BCV (Smith et al., 2016). Following replication, some of the bacteria associate with an autophagosome-like compartment, termed the aBCV (Starr et al., 2012). Formation of the aBCV requires Beclin1 and ULK1 but not the autophagy elongation proteins ATG4B, ATG5, ATG7, ATG14L, or LC3B (Starr et al., 2012). Generation of the aBCV promotes completion of the *Brucella* lifecycle by promoting bacterial egress and cell-to-cell spread (Starr et al., 2012). However, further work is needed to elucidate the cellular signals that trigger aBCV formation and the bacterial factors that are involved in this process.

Rab2, a small GTPase involved in retrograde trafficking, is important for formation of the BCV and *Brucella* replication (Fugier et al., 2009). In a yeast-2-hybrid screen to identify *Brucella*-host interactions, RicA was shown to specifically interact with Rab2 (Table 1). RicA preferentially interacts with the GDP-bound form of Rab2, however it does not appear to have GEF activity (de Barsy et al., 2011). While *ricA* deletion does not significantly impact intracellular replication, RicA is needed for recruitment of Rab2 and appropriate trafficking of the BCV (de Barsy et al., 2011), highlighting the importance of retrograde trafficking to the development of the BCV. Multiple VirB T4SS effector proteins (BspA, BspB, and BspF) impair host protein secretion when ectopically produced (Myeni et al., 2013). While the functions of BspA and BspF are unknown, BspB co-precipitates with a number of subunits of the COG complex (COG1, COG2, COG3, COG4, COG5, and COG7). The COG complex is a tethering platform associated with the Golgi apparatus that mediates docking and fusion events within the Golgi apparatus in addition to playing a role in retrograde trafficking (Miller et al., 2017). BspB promotes rBCV biogenesis and intracellular replication by invoking changes in anterograde and retrograde transport, resulting in redistribution of COG-dependent vesicles to the BCV (Miller et al., 2017). The fact that multiple proteins impair the secretory pathway highlights the importance of remodeling this pathway during *Brucella* infection. However, further work is needed to elicit the role BspA and BspF play in modulating host secretion.

## *Salmonella enterica* serovar typhimurium

**Figure 4:**
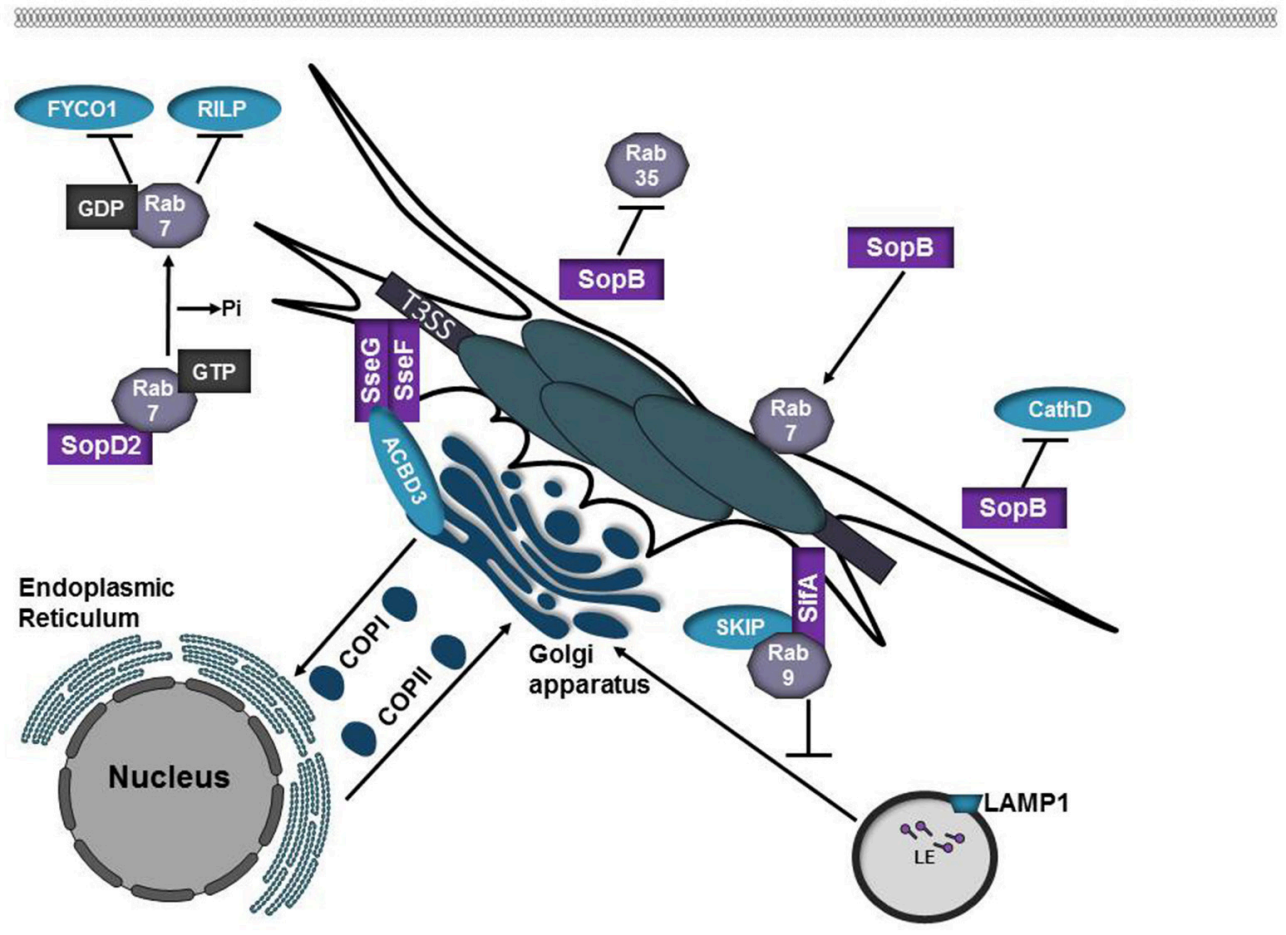
Salmonella SPI-2 effector proteins participate in SCV formation. SopB is phosphoinositide phosphatase that alters the membrane surface charge of the SCV. This prevents targeting by Rab35 while promoting recruitment of Rab7. SopB also inhibits targeting of the lysosomal protease Cathepsin D to the SCV. SopD2 is a Rab7 GAP. Inactivation of Rab7 by SopD2 prevents interactions with the Rab7 effectors RILP and FYCO1. SifA is inserted into the SCV membrane where it interacts with SKIP/PLEKHM2. SifA-SKIP sequesters Rab9 at the SCV, inhibiting retrograde trafficking of mannose-phosphate receptors (MPRs) to the *trans-*Golgi network. SseF and SseG are integral membrane effector proteins that form a trimolecular complex with ACBD3 to anchor the SCV to the Golgi apparatus.

*Salmonella enterica* serovar Typhimurium is the leading cause of foodborne illness worldwide. Infection typically manifests as a self-limiting gastrointestinal disease, however in immunocompromised individuals the infection can develop into a systemic disease (Anderson and Kendall, 2017). Manipulation of host cell function in *Salmonella enterica* serovar Typhimurium is mediated by over 30 effector proteins that are delivered into the host cell by two type III secretion systems (T3SS), encoded on two *Salmonella* pathogenicity islands (SPI-1 or SPI-2; Galan and Curtiss, 1989; Shea et al., 1996). SPI-1 translocates effectors that promote invasion of non-phagocytic cells and modulates host cell signaling pathways whereas SPI-2 is expressed following internalization and SPI-2 effectors manipulate host vesicular trafficking pathways to promote intracellular survival (Jennings et al., 2017). Following internalization, *Salmonella* replicates in a *Salmonella*-containing vacuole (SCV) that matures by trafficking through the endocytic pathway and interacts with the secretory pathway (Vogels et al., 2011; Jennings et al., 2017; Figure 2). Despite the fact that specific lysosomal membrane glycoproteins are present in the SCV, markers such as mannose-6-phosphate receptors (MPRs) and cathepsin D are absent (Rathman et al., 1997; McGourty et al., 2012), indicating that SCV interactions with lysosomes are highly controlled. The effector protein SopB contributes to invasion and is required for the recruitment of Rab5 and the PI 3-kinase Vps34 to the SCV as well as acquisition of LAMP1 from non-lysosomal sources (Mallo et al., 2008; Bakowski et al., 2010). SopB can also function as a phosphoinositide phosphatase that reduces the levels of phosphatidylinositol-4,5-bisphosphate [PI(4,5)P_2_] and phosphatidylserine (PS) in the SCV, altering the membrane surface charge (Bakowski et al., 2010). Changes in the surface charge of the SCV membrane promotes lysosomal avoidance by inhibiting targeting by specific Rab GTPases, such as Rab35 (Bakowski et al., 2010; Figure 4, Table 1).

The SPI-2 effector SopD2 has been shown to modulate the function of multiple Rab GTPases (D'Costa et al., 2015; Spanò et al., 2016; Teo et al., 2017). SopD2 disrupts endocytic trafficking by acting as a Rab7 GAP, which prevents interactions with the Rab7 effector RILP and the kinesin-binding protein FYCO1 (D'Costa et al., 2015). SopD2 also functions as a Rab32 GAP. Rab32 has been shown to restrict *Salmonella* Typhi intracellular replication (Spanò and Galán, 2012). In *Salmonella* Typhimurium, SopD2 in conjugation with the GtgE protease acts to prevent Rab32 accumulation on the SCV presumably, to prevent delivery of anti-microbial compounds to the SCV (Spanò et al., 2016). Multiple effectors, including SopD2 and GtgE, are absent or are pseudogenes in *Salmonella* Typhi and the loss of these effectors may partially explain the differences in *Salmonella enterica* serovar host selectivity and the inability of these serovars to avoid specific antimicrobial pathways such as those that depend on Rab32.

The SPI-2 effector SifA is required for maintenance of vacuolar integrity (Beuzón et al., 2000) and formation of tubules, termed *Salmonella-*induced filaments (SIFs) that project from the SCV (Garcia-del Portillo et al., 1993). A C-terminal CAAX motif within SifA is prenylated by eukaryotic host geranylgeranyl transferase I, allowing insertion of SifA into the SCV membrane (Reinicke et al., 2005). From the vacuolar membrane, SifA interacts with SKIP/PLEKHM2 to control kinesin motor activity and SIF formation (Boucrot et al., 2005). Binding of SifA to SKIP allows for the bacteria to sequester Rab9 at the SCV, disrupting retrograde trafficking of MPRs to the *trans-*Golgi network, thus reducing lysosomal potency (McGourty et al., 2012). Fusion between SCVs and lysosomes is presumed to supply membrane and nutrients to the SCV and bacteria within (Jennings et al., 2017).

SseF and SseG are integral membrane effector proteins that heterodimerize and localize to the SCV and SIFs following translocation via SPI-2. Whereas SCVs harboring wild-type bacteria are immobile and remain in close proximity to the Golgi, mutation of SseF or SseG results in SCVs that are dispersed throughout the cytosol and are highly mobile (Salcedo and Holden, 2003; Abrahams et al., 2006; Deiwick et al., 2006). SseF and SseG bind Golgi protein acyl-CoA binding domain containing 3 (ACBD3) to form a trimolecular complex to tether the SCV to the Golgi (Yu et al., 2016). Tethering of the SCV to the Golgi might facilitate acquisition of membrane and nutrients.

In a screen to identify host factors that control SCV positioning, Mota et al. demonstrated that secretory carrier membrane proteins (SCAMPS) 2 and 3 play an integral role in maintaining SCV positioning and depletion of SCAMP2 or 3 results in dispersion of the SCV within the host cell (Mota et al., 2009). Infection of host cells with *Salmonella* induces the formation of SCAMP3 tubules that overlap with SIFs, but unlike SIFs these tubules lack endosomal proteins. While several SPI-2 effector proteins co-localize with SCAMP3 (Mota et al., 2009), whether these effectors play a role in the formation of SCAMP3 tubules requires further study.

## Legionella pneumophila

**Figure 5:**
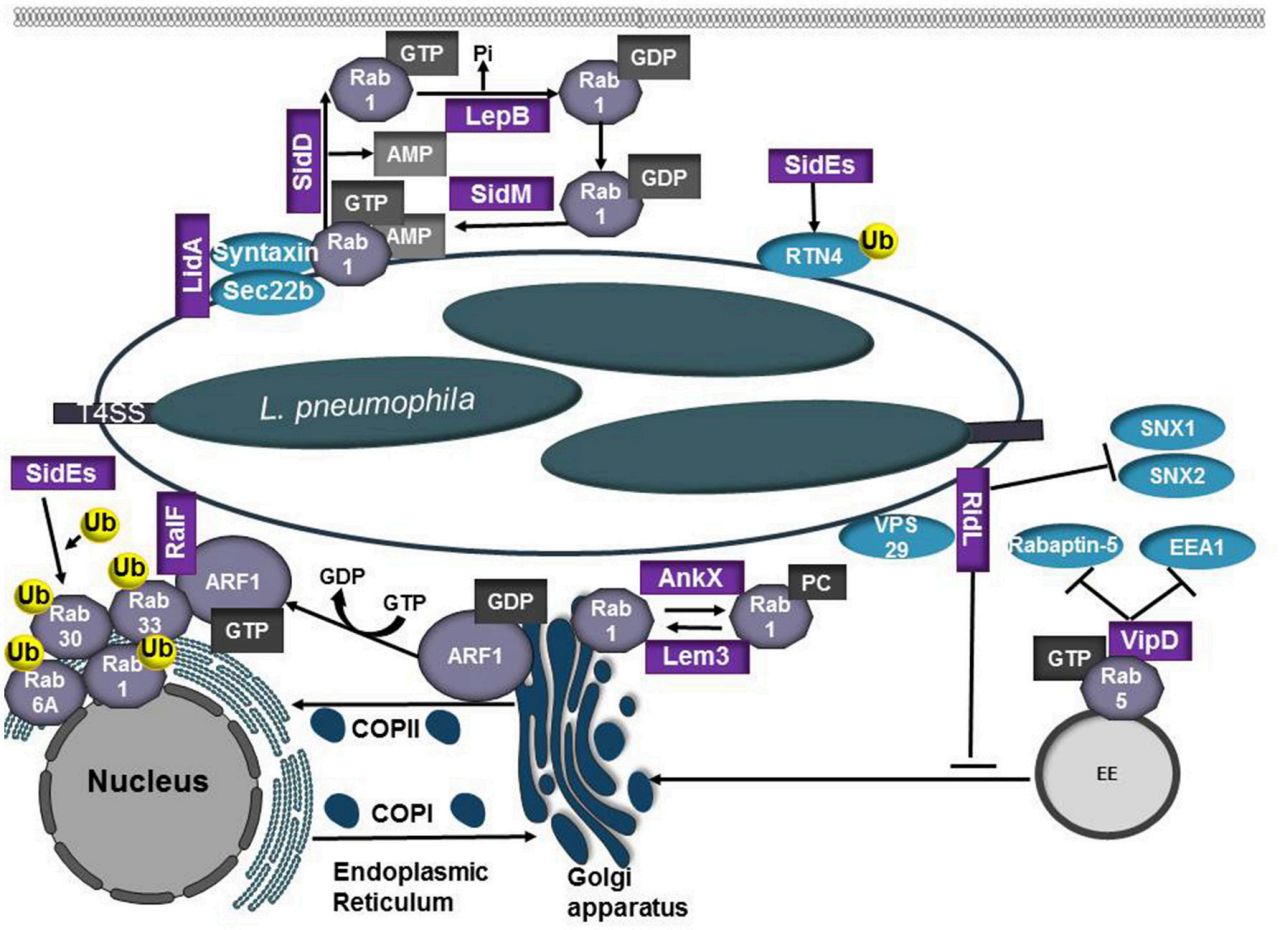
Role of *L. pneumophilia* T4SS effector proteins in modulation of the endocytic and secretory pathways. *L. pneumophila* recruits the small GTPases Rab1 and Arf1 to the LCV membrane to co-opt ER-Golgi vesicle trafficking. RalF and SidM/DrrA are Arf1 and Rab1 GEFs, respectively. SidM/DrrA can covalently modify Rab1 through AMPylation whereas SidD functions as a deAMPylase to remove the AMP residue, making Rab1 susceptible to inactivation by the bacterial GAP LepB. Activated Rab1 promotes binding of LidA, which cooperates with SidM/DrrA to promote tethering and fusion between the LCV and ER-derived vesicles. AnkX and Lem3 modify Rab1 activity through phosphorylcholination (PCylation) or by removal of the phosphorylcholine moiety, respectively. The SidE family of T4SS effectors (SidE, SdeA, SdeB, SdeC) ubiquitinate Rab1, Rab6, Rab30, and Rab33b. SidE effectors also ubiquitinate reticulon 4 (RTN4) to induce ER rearrangements and promote RTN4 recruitment to the LCV. RidL binds PtdIns(3)P and the retromer subunit VPS29, inhibiting SNX 1 and 2 binding to the LCV. VipD prevents endosomal maturation by binding GTP-bound Rab5 and Rab22, inhibiting subsequent interactions with the Rab effectors Rabaptin-5 and EEA1.

*Legionella pneumophila* is a facultative intracellular opportunistic pathogen that causes Legionnaires' disease, a severe form of pneumonia. In both amoebas and human alveolar macrophages, *L. pneumophilia* replicates in a parasitophorous vacuole termed the *Legionella*-containing vacuole (LCV; Swanson and Isberg, 1995; Figure 2). Biogenesis of the LCV requires translocation of Dot/Icm T4SS effector proteins (Berger and Isberg, 1993; Brand et al., 1994). While the precise details of how *L. pneumophila* evades endocytic maturation are unknown, the type IV effector protein VipD appears to play a role in this process. VipD binds GTP-bound Rab5 and Rab22, preventing subsequent interactions with the Rab effectors Rabaptin-5 and EEA1 (Ku et al., 2012; Figure 5, Table 1). Upon binding to Rab5, the phospholipase A1 activity of VipD is activated, which alters the protein and lipid composition of the endosomal membrane and prevents further endosomal maturation (Gaspar and Machner, 2014; Lucas et al., 2014).

Following internalization into a host cell, *L. pneumophila* recruits the small GTPases Rab1 and Arf1 to the LCV membrane to gain control of ER-Golgi vesicle trafficking. The *L. pneumophila* T4SS effector RalF functions as a GEF for multiple ADP ribosylation factor (Arf) GTPases (Nagai et al., 2002). Recruitment of Arf1 to the LCV and its subsequent activation by RalF may promote LCV expansion by facilitating recruitment and recycling of ER-derived vesicles. Rab1 recruitment to the *Legionella*-containing vacuole is mediated by the T4SS effector SidM/DrrA, which binds to PtdIns4P in the LCV membrane (Brombacher et al., 2009). SidM/DrrA is a multifunctional effector with a high affinity for GDP-bound Rab1. SidM/DrrA promotes activation of Rab1 by functioning as a GEF (Machner and Isberg, 2006; Murata et al., 2006) and can covalently modify Rab1 through AMPylation of Tyr77, which prevents access by GAPs and promotes the maintenance of Rab1 in an active, GTP-bound state (Müller et al., 2010). Rab1 activation by SidM/DrrA stimulates association with syntaxins and Sec22b. Noncanonical pairing of the ER-derived SNARE protein Sec22b with plasma membrane syntaxins 2, 3, and 4 on the LCV allows for fusion of ER-derived vesicles with the PM-derived LCV (Arasaki et al., 2012). Activated Rab1 on the LCV promotes binding of LidA, which cooperates with SidM/DrrA to enhance tethering and fusion of ER-derived vesicles to the LCV (Machner and Isberg, 2006). SidD functions as a deAMPylase and removes the AMP residue (Neunuebel et al., 2011; Tan and Luo, 2011), making Rab1 susceptible to inactivation by the bacterial GAP LepB (Ingmundson et al., 2007). Inactivation of Rab1 results in disassociation of the Rab GTPase from the LCV. The *L. pnuemophila* effectors AnkX and Lem3 also control Rab1 activity through phosphorylcholination (PCylation) or de-PCylation, respectively (Tan et al., 2011; Goody et al., 2012). It was recently demonstrated that AnkX perturbs transferrin recycling and prevents accumulation of lysosomal markers on the LCV, both of which requires AnkX phosphocholination activity, providing keen insight into the potential functional consequence of this covalent modification (Allgood et al., 2017). While both AMPylated and PCylated Rab1 have been isolated from *L. pneumophila* infected cells, Rab1 possessing both modifications has not been observed (Mukherjee et al., 2011). Further research is needed to resolve the roles these specific modifications play in maturation of the LCV and whether these modifications are necessary during specific stages of the infection cycle.

Rab1, Rab6, Rab30, and Rab33b are ubiquitinated by members of the SidE family of T4SS effectors (SidE, SdeA, SdeB, SdeC). Interestingly, ubiquitination of these proteins does not require host E1 or E2 enzymes, representing a method of ubiquitination that is unique to *L. pneumophila* (Qiu et al., 2016; Qiu and Luo, 2017). Ubiquitination of Rabs impacts GTPase activity but does not result in degradation of the Rab GTPase (Qiu et al., 2016). The SidE family of effectors also ubiquitylates reticulon 4 (RTN4) to induce ER rearrangements and promotes RTN4 recruitment to the LCV (Kotewicz et al., 2017), however the significance of this recruitment is unknown.

Retrograde transport is important for returning resident proteins and receptors for another round of cargo selection and maintenance of homeostasis within the cell. The multiprotein retromer complex plays an integral role in cargo transport from endosomes to the *trans-*Golgi network and is recruited to target membranes by GTP-bound Rab7 (Personnic et al., 2016). This complex is composed of vacuolar protein sorting (VPS) 26, VPS29, and VPS35 that associate with sorting nexins (SNX) 1 or SNX2 and SNX5 or SNX6. The *L. pneumophila* T4SS effector RidL can bind to the retromer subunit VPS29 and PtdIns(3)P and potentially modulates the activity of the retromer complex. Mutation of *ridL* results in decreased *L. pneumophila* replication in macrophages, LAMP1 accumulation on the LCV, as well as recruitment of retrograde cargo receptors (Vps10 and CIMPR) and SNX1 and 2 (Finsel et al., 2013). siRNA knockdown of retromer components significantly increases *L. pneumophila* replication in mammalian cells, suggesting retrograde trafficking pathways restrict *L. pneumophila* intracellular replication (Finsel et al., 2013). RidL binding to PtdIns(3)P may promote sufficient competition for SNX1 and 2 binding, which in turn promotes removal of retromer components from the LCV (Finsel et al., 2013). Preventing association with the retromomer complex may be a strategy used by *L. pneumophila* to promote formation of a non-lysosomal vacuole that is permissive for replication.

## Chlamydia trachomatis

**Figure 6:**
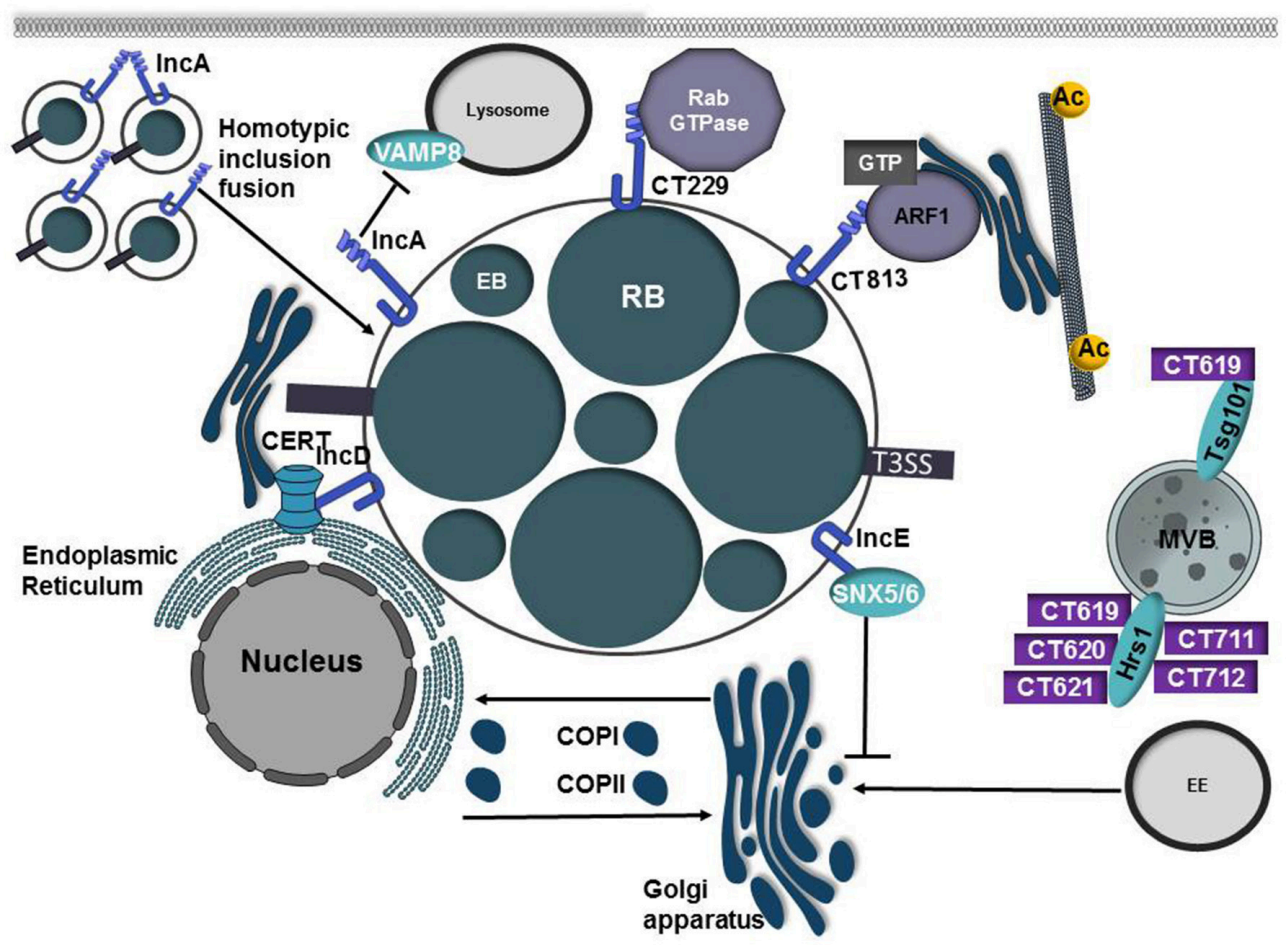
Inclusion membrane proteins and secreted effectors from *C. trachomatis* co-opt host vesicular trafficking pathways to promote inclusion development. *Chlamydia trachomatis* replicates in a parasitophorous vacuole, termed the inclusion. Early in the infection cycle the inclusion is modified through the incorporation of type III secreted effector proteins known as inclusion membrane proteins (Incs). IncA possesses 2 coiled-coil domains that are homologous to eukaryotic SNARE motifs and these domains are required to mediate homotypic fusion of inclusions. IncA is also able to interact with VAMP8 on lysosomes and may prevent inclusion fusion with lysosomes. *C. trachomatis* manipulates and recruits Rab and Arf GTPases to the inclusion membrane through interactions with the inclusion membrane proteins CT229 and CT813, respectively. While the mechanism of CT229-Rab interactions is ill-defined, CT813 is presumed to modulate Arf GTPases to control Golgi ministack positioning by regulating microtubule posttranslational modification. IncE interacts with SNX5/6, components of the retromer complex, to disrupt trafficking to the *trans-*Golgi network and to promote chlamydial infection. To acquire the sphingomyelin precursor ceramide, IncD interacts with the Pleckstrin homology (PH) domain of CERT allowing ceramide to be directly transferred from the ER to the inclusion membrane. *C. trachomatis* also secretes a large repertoire of type III proteins into the eukaryotic cells. CT619, CT620, CT621, CT711, CT712 are type III substrates that interact with Hrs and/or Tsg101, components of the ESCRT pathway.

*Chlamydia trachomatis* is the leading cause of blinding trachoma and one of the most prevalent sexually transmitted infections caused by a bacterium (Elwell et al., 2016). *C. trachomatis* replicates in the epithelium of the urethra in men and the endocervix in women, resulting in inflammation and edema. In women, 15–40% of infections spread to the upper genital tract, resulting in pelvic inflammatory disease (PID), ectopic pregnancy, and infertility (Elwell et al., 2016). All chlamydiae share a biphasic developmental cycle in which they transition from the extracellular, infectious elementary body (EB) to the non-infectious reticulate body (RB) in a membrane-bound vacuole termed the inclusion (Elwell et al., 2016; Figure 2). At all stages of the developmental cycle, chlamydia translocates bacterial effector proteins into the host cell using a type III secretion system. While a subset of these proteins localize within the host cell (Chellas-Gery et al., 2007; Hower et al., 2009; Pennini et al., 2010), an additional subset of proteins, termed inclusion membrane proteins (Incs) bind to the inclusion membrane and mediate crucial host-pathogen interactions (Weber et al., 2015). Intracellular survival of chlamydia requires the inclusion to inhibit fusion with lysosomes while promoting fusion with other compartments such as exocytic vesicles. In eukaryotic cells, SNARE proteins present on the target organelle (t-SNARE) and vesicle (v-SNARE) assemble into a four-helix bundle to bring membranes into close proximity and drive membrane fusion (Nickel et al., 1999). *C. trachomatis* encodes at least three Inc proteins that possess SNARE-like domains (SLD); IncA (CT119), InaC (CT813), and IPAM (CT223) (Delevoye et al., 2008). IncA possesses 2 coiled-coil domains that are homologous to eukaryotic SNARE motifs and full-length SLD1 and part of SLD2 are required to mediate homotypic fusion of inclusions (Ronzone and Paumet, 2013; Ronzone et al., 2014; Weber et al., 2016c; Figure 6, Table 1). Both IncA and CT813 co-precipitate with several VAMPs and IncA is able to inhibit fusion with compartments possessing VAMP3, VAMP7, or VAMP8 (Delevoye et al., 2008). While IncA is not expressed until 10 h post-infection (Hackstadt et al., 1999; Belland et al., 2003), IncA's ability to inhibit fusion and to interact with VAMP8 on lysosomes (Delevoye et al., 2008; Paumet et al., 2009) suggests that IncA may prevent inclusion fusion with lysosomes later in the developmental cycle. Inhibition of bacterial protein synthesis with chloroamphenicol results in delayed fusion of vesicles containing EBs with lysosomes, suggesting that some inherent factor on the EB acts early to subvert this host defense mechanism (Scidmore et al., 2003). This delay in lysosomal fusion has led to a model in which initially an intrinsic property of the EBs cause minimal interactions with the endocytic pathway, however at later stages of infection (~8 h) lysosomal evasion requires bacterial protein synthesis and modification of the inclusion membrane by the incorporation of Inc proteins (Fields and Hackstadt, 2002).

Like other intracellular pathogens, *C. trachomatis* manipulates Arf and Rab GTPases, presumably to redirect host vesicles to the inclusion for acquisition of lipids and other nutrients. Rab GTPases are recruited to the inclusion membrane, some in a species-specific manner (Rzomp et al., 2003; Aeberhard et al., 2015). Rab11, Rab4, and Rab1 are recruited to the inclusions of multiple chlamydial species whereas Rab6 recruitment is restricted to *C. trachomatis* inclusions and Rab6 is not recruited to *C. pneumoniae* or *C. muridarum* inclusions (Rzomp et al., 2003). CT229 from *C. trachomatis* binds multiple Rab GTPases involved in ER to Golgi transport, retrograde transport, exocytosis, and lipid droplet formation (Rzomp et al., 2006; Mirrashidi et al., 2015; Sixt et al., 2017). Recent studies have shown that CT229 is essential for intracellular replication and the absence of CT229 triggers premature host cell death (Sixt et al., 2017; Weber et al., 2017). Strikingly the absence of other Inc proteins (IncC and CT383) similarly results in defects in intracellular replication, premature inclusion lysis, activation of the STING pathway, and induction of a type of intrinsic apoptosis that does not require caspase-3 activation (Weber et al., 2017). Further work is needed to resolve whether the phenotype of these Inc mutants is a general consequence related to premature inclusion lysis or if the induction of host cell death is related to potential effector function. Interestingly other chlamydial species that do not encode a CT229 homolog are still able to recruit Rab GTPases to the inclusion, suggesting additional bacterial proteins may be involved in Rab recruitment (Rzomp et al., 2003, 2006). Indeed Cpn0585, a *C. pneumoniae* Inc protein interacts with Rab1, Rab10, and Rab11 (Cortes et al., 2007) highlighting the importance of Rab subversion by multiple chlamydial species.

Following uptake by the host cell, the chlamydial inclusion traffics along microtubules to the microtubule organizing center (MTOC) where it undergoes extensive interactions with the Golgi (Grieshaber et al., 2003; Elwell et al., 2016). During *C. trachomatis* infection, the Golgi is fragmented into ministacks that are repositioned around the inclusion to facilitate access to lipids and nutrients (Heuer et al., 2009). The positioning of Golgi ministacks is controlled by a cage of microtubules enriched in acetylated and detyrosinated tubulin (Al-Zeer et al., 2014). Microtubule depolymerization of post-translationally modified microtubules promotes Golgi ministack dispersal (Al-Zeer et al., 2014). Arf GTPases regulate the structure of the Golgi complex by regulating the lipid concentration, cisternal maturation, and vesicle trafficking (Donaldson and Jackson, 2011). The inclusion membrane protein CT813 (lnaC) was recently shown to bind and recruit Arf1 and Arf4 to the inclusion membrane (Kokes et al., 2015; Wesolowski et al., 2017). While CT813 induces Arf1 activation it does not possess GEF activity, suggesting it may recruit an Arf GEF to promote Arf activation (Wesolowski et al., 2017). Mutation of CT813 or depletion of Arf1 or Arf4 prevents Golgi dispersal and decreases the amount of detyrosinated or aceylated alpha-tubulin, suggesting that CT813 hijacks Arf GTPases to control Golgi positioning during infection (Wesolowski et al., 2017). The chlamydial inclusion is also encased in an actin cage and loss of CT813 diminishes actin cage assembly around the inclusion (Kokes et al., 2015), however the mechanism(s) by which CT813 regulates both cytoskeletal components is unknown.

In a study to map the host-Inc interactome, an interaction between the inclusion membrane protein IncE and SNX5/6, a component of the retromer complex, was identified (Mirrashidi et al., 2015). Infection with *C. trachomatis* causes relocalization of SNXs from endosomes to the inclusion membrane and induces inclusion tubulation (Aeberhard et al., 2015; Mirrashidi et al., 2015). Similar to *L. pneumophila*, knockdown of retromer components enhances *C. trachomatis* replication, suggesting the retromer complex controls infection (Mirrashidi et al., 2015). By recruiting retromer to the inclusion, IncE relieves the restriction placed on the pathogen and allows the bacteria to disrupt trafficking to the *trans*-Golgi network (Mirrashidi et al., 2015; Elwell et al., 2017). However, whether IncE is necessary and sufficient to induce retromer relocalization remains unknown due to the thus far unsuccessful attempts to isolate an IncE mutant (Kokes et al., 2015).

In addition to acquiring nutrients by exploiting host vesicular trafficking pathways, *C. trachomatis* hijacks non-vesicular ER-TGN transport to acquire ceramide, a sphingomyelin precursor. Host lipids including sphingomyelin, cholesterol, phosphatidylcholine, and phosphatidylinositol are incorporated into the bacterial cell (Hackstadt et al., 1996; Wylie et al., 1997; Carabeo et al., 2003). During chlamydial infection the inclusion membrane protein IncV (CT005) interacts with VapA and VapB on the endoplasmic reticulum to tether the inclusion to the ER (Stanhope et al., 2017). The formation of these ER-inclusion membrane contact sites (MCS) is believed to promote lipid transfer directly to the bacteria (Derré et al., 2011). The ceramide transport protein (CERT) localizes to ER-Golgi contact sites and promotes transfer of ceramide from the ER to the Golgi (Derré et al., 2011). The inclusion membrane protein IncD interacts with the Pleckstrin homology (PH) domain of CERT, allowing the sphingomyelin precursor ceramide to be directly transferred from the ER to the inclusion membrane (Derré et al., 2011). Both sphingomyelin and CERT are important for chlamydial infection and depletion of either reduces bacterial replication and results in smaller inclusions (Derré et al., 2011). Formation of ER-inclusion MCSs allows for direct import of ceramide and synthesis of sphingomyelin at the inclusion membrane.

While a large number of *C. trachomatis* T3SS substrates localize to the inclusion membrane, an additional subset of T3SS proteins are predicted to be secreted into the host cell cytosol (Subtil et al., 2005; Muschiol et al., 2011; Pais et al., 2013; da Cunha et al., 2014). CT619, CT620, CT621, CT711, CT712 are type III substrates that possess a DUF582 domain, which is found in all pathogenic strains of chlamydia (Muschiol et al., 2011). Using the DUF582 domain as bait in a yeast-2-hybrid screen, Hrs a component of the endosomal sorting complexes required for transport (ESCRT) was identified as capable of interacting with the domain from each secreted effector except CT621 (Vromman et al., 2016). The N-terminus of CT619 was also found to interact with the ESCRT-1 protein Tsg101. While this study suggests that several secreted effectors interact with the ESCRT pathway, the biological relevance of this interaction remains unknown as disruption of Hrs, Tsg101, or other essential components of the ESCRT pathway did not impair chlamydial growth.

## Orientia tsutsugamushi

*Orientia tsutsugamushi* is an obligate intracellular bacterium that is the causative agent of scrub typhus, a potentially fatal disease that is endemic to the Asia-Pacific region. *O. tsutsugamushi* is transmitted to humans via the bite of an infected trombiculid mite (Valbuena and Walker, 2012) after which the bacteria invades the dermis, causing an inflammatory lesion known as an eschar. Infected leukocytes migrate from the bite site to local lymph nodes where it spreads to the peripheral vascular system to ultimately infect endothelial cells of the skin and major organs (Paris et al., 2012). *O. tsutsugamushi* is internalized by clathrin-dependent endocytosis and associates with early and late endosomes as evident by co-localization with EEA1 and LAMP2, respectively (Chu et al., 2006; Figure 2). *O. tsutsugamushi* is released into the cytoplasm where it moves along microtubules to the MTOC (Kim et al., 2001) where the bacteria replicate by binary fission in the cytosol adjacent to the ER and Golgi (Ge and Rikihisa, 2011). How *O. tsutsugamushi* escapes the phagosome is unknown, however it encodes a hemolysin, tlyC and a phospholipase D (Ge and Rikihisa, 2011). Whether either of these proteins is involved in bacterial escape warrants further study.

The ankyrin repeat domain is a 33-residue eukaryotic motif involved in mediating protein-protein interactions for numerous host cell processes including transcription, cell cycle regulation, signal transduction, and cytoskeletal rearrangements (Voth et al., 2009). To facilitate interactions with host proteins, many intracellular bacteria including *C. burnetii* (Pan et al., 2008; Voth et al., 2009), *L. pneumophila* (Pan et al., 2008), and *Anaplasma phagocytophilum* (Caturegli et al., 2000) encode Ank proteins that are secreted into the host cell to promote subversion of host cell processes. *O. tsutsugamushi* Ikeda possesses 47 Ank open reading frames (ORFs; Nakayama et al., 2008), the largest number of any bacterial species. While several of the Ank ORFs are pseudogenes or identical or near identical to other Anks, 20 unique Ank ORFs are expressed during infection and are translocated by a type I secretion system (T1SS; VieBrock et al., 2015). Ank9 possesses seven ankyrin repeats and a C-terminal F-box that is required for interactions with S-phase kinase associated protein 1 (SKP1; Beyer et al., 2015). Ank9 also encodes an N-terminal GRIP-like domain that is required for localization to the Golgi. At the Golgi apparatus, Ank9 interacts with coatomer protein complex subunit beta 2 (COPB2) to co-opt COPI-mediated retrograde trafficking to the ER. Subversion of retrograde trafficking by Ank9 induces ATF4-mediated unfolded protein response (UPR), ultimately disrupting host protein secretion (Beyer et al., 2017; Table 1). During *Orientia* infection, the ER is distended and the Golgi is perturbed. While the exact mechanism of how this occurs and the benefit to *Orientia* is unknown, it is possible that at least part of this is dependent on Ank9.

## Conclusions

Obligate and facultative intracellular bacteria have developed sophisticated strategies to modulate host endocytic and secretory trafficking to promote formation of their unique replicative niches. The adaption of genetic tools for manipulation of obligate and facultative intracellular pathogens has substantially enhanced our understanding of host-pathogen interactions and effector function. While great strides have been made toward understanding how effector proteins manipulate host processes to redirect membrane and nutrients to the parasitophorous vacuoles, the function of most effector proteins still remains ill-defined and genetic manipulation of some of these organism presents specific challenges. Large-scale screens to identify putative binding partners of ectopically produced type III secreted effectors (Mirrashidi et al., 2015) or yeast-2-hybrid screening of type IV secreted effectors (Wallqvist et al., 2017) has identified potential interacting partners for many previously uncharacterized effector proteins. While these seminal studies will serve as a useful starting point for elucidating effector function of uncharacterized secretion substrates, many of these interactions still require validation. Future studies looking at these interactions in the context of infection will be crucial for validating these interactions. As the genetic toolbox expands for these organisms, large-scale screening for interacting partners in infected cells will be quite useful to identify interactions between effector proteins as well as with the host proteins.

## Author contributions

All authors listed have made a substantial, direct, and intellectual contribution to the work, and approved it for publication.

### Conflict of interest statement

The authors declare that the research was conducted in the absence of any commercial or financial relationships that could be construed as a potential conflict of interest.
